# Life- and person-centred help in Mecklenburg-Western Pomerania, Germany (DelpHi): study protocol for a randomised controlled trial

**DOI:** 10.1186/1745-6215-13-56

**Published:** 2012-05-10

**Authors:** Jochen René Thyrian, Thomas Fiß, Adina Dreier, Georgia Böwing, Aniela Angelow, Sven Lueke, Stefan Teipel, Steffen Fleßa, Hans Jörgen Grabe, Harald Jürgen Freyberger, Wolfgang Hoffmann

**Affiliations:** 1 German Center for Neurodegenerative Diseases (DZNE) Greifswald, Ellernholzstrasse 1-2, Greifswald, D-17487, Germany; 2 Department Epidemiology of Health Care and Community Health, Institute for Community Medicine, Ernst-Moritz-Arndt-University, Ellernholzstrasse 1-2, Greifswald, D-17487, Germany; 3 Department of Family Medicine, Institute for Community Medicine, Ellernholzstrasse 1-2, Greifswald, D-17487, Germany; 4 Institute for Business Administration and Health Care Management, Ernst-Moritz-Arndt-University, Friedrich-Loeffler-Strasse 70, Greifswald, D-17489, Germany; 5 Department of Psychiatry and Psychotherapy, University of Rostock, Gehlsheimer Strasse 20, Rostock, D-18471, Germany; 6 German Center for Neurodegenerative Diseases (DZNE) Rostock, Gehlsheimer Strasse 20, Rostock, D-18471, Germany; 7 Department of Psychiatry and Psychotherapy, University of Greifswald, Ellernholzstrasse 1-2, D-17489, Greifswald, Germany; 8 German Center for Neurodegenerative Diseases (DZNE) Rostock/Greifswald, Ellernholzstrasse 1-2, D-Greifswald, 17487, Germany

**Keywords:** Dementia care, Population-based, Care management, Intervention

## Abstract

**Background:**

The provision of appropriate medical and nursing care for people with dementia is a major challenge for the healthcare system in Germany. New models of healthcare provision need to be developed, tested and implemented on the population level. Trials in which collaborative care for dementia in the primary care setting were studied have demonstrated its effectiveness. These studies have been conducted in different healthcare systems, however, so it is unclear whether these results extend to the specific context of the German healthcare system.

The objective of this population-based intervention trial in the primary care setting is to test the efficacy and efficiency of implementing a subsidiary support system on a population level for persons with dementia who live at home.

**Methods and study design:**

The study was designed to assemble a general physician-based epidemiological cohort of people above the age of 70 who live at home (DelpHi cohort). These people are screened for eligibility to participate in a trial of dementia care management (DelpHi trial). The trial is a cluster-randomised, controlled intervention trial with two arms (intervention and control) designed to test the efficacy and efficiency of implementing a subsidiary support system for persons with dementia who live at home. This subsidiary support system is initiated and coordinated by a dementia care manager: a nurse with dementia-specific qualifications who delivers the intervention according to a systematic, detailed protocol. The primary outcome is quality of life and healthcare for patients with dementia and their caregivers. This is a multidimensional outcome with a focus on four dimensions: (1) quality of life, (2) caregiver burden, (3) behavioural and psychological symptoms of dementia and (4) pharmacotherapy with an antidementia drug and prevention or suspension of potentially inappropriate medication. Secondary outcomes include the assessment of dementia syndromes, activities of daily living, social support health status, utilisation of health care resources and medication.

**Discussion:**

The results will provide evidence for specific needs in ambulatory care for persons with dementia and will show effective ways to meet those needs. Qualification requirements will be evaluated, and the results will help to modify existing guidelines and treatment paths.

**Trial registration:**

NCT01401582

## Background

The provision of appropriate medical and nursing care for people with dementia is a major challenge for the healthcare system in Germany. As a result of demographic changes, the overall population will decrease over the next few decades and there will be an increase in the number of people above the age of 65 [1]. These changes will be accompanied by an increase of people with age-associated illnesses such as diabetes, hypertension and dementia. The global prevalence of dementia in this age group was estimated to be as high as 24 million in 2001, and, according to current estimates, it is predicted to double every 20 years and affect more than 80 million people worldwide by 2040. The estimated annual incidence and prevalence of Alzheimer disease, the most frequent cause of dementia in the elderly, dramatically rise concomitantly with age. Incidence rates from approximately 0.4% in people ages 65 to 69 up to nearly 10% in people over 90 years of age have been reported, along with a prevalence from approximately 2% in people ages 65 to 69 years up to more than 25% in those over 90 years of age [2]. In Germany, this estimate corresponds to a prevalence of approximately 1.1 million people and an annual incidence of 250,000 new patients [3]. Analyses and prognoses for the federal state of Mecklenburg-Western Pomerania reveal that the number of people with dementia will increase from about 80% in 2005 to approximately 91% in 2030 [4]. To meet the challenges associated with these trends, new models of healthcare provision need to be developed, tested and implemented on the population level.

A current review describes the status of dementia research and care in Germany [5]. According to that review, there is an urgent need for population-based research to meet the challenges of (1) early identification in the sense of widespread availability of early diagnostic testing, including the proven effectiveness and early initiation of therapy where appropriate; (2) multimorbidity, with dementia’s interfering with the treatment of other diseases, as well as comorbidity that aggravates the clinical course of dementia; (3) integration of multiprofessional strategies to develop comprehensive treatment and management of persons with dementia in the existing healthcare system; and (4) consequently addressing caregiver burden. The epidemiology of healthcare research has provided valuable results to support ways to address each of these needs separately. However, population-based research targeting this complex situation in a comprehensive, integrated way still needs to be carried out.

It is well-known that the population-based efficacy of early intervention is dependent on the effectiveness of the intervention itself, the availability of the intervention for all people affected and the utilisation of the intervention by these people [6]. Current data show, however, that many persons with dementia are not diagnosed at all or are diagnosed only at a later, already clinically advanced stage of the disease. In a German population-based study of persons with dementia, 51% of participants had received the diagnosis “dementia” from their general practitioners (GPs) (unpublished data, JR Thyrian). Dementia was overrepresented in depressed patients and in those with hearing impairments and restricted mobility. Persons living in single-person households, however, were often underdiagnosed with dementia [7]. The risks and benefits of systematic screening for cognitive impairment have been discussed with much controversy in the international literature, and no data exist about systematic screening for the purpose of dementia intervention in Germany [8-11].

Multimorbidity is a challenge unmet in Germany. Subjects with dementia frequently have other age-associated illnesses. In a Swedish population-based study, investigators found that only 34% of all persons with dementia did not have comorbid diseases. Additionally, dementia is a risk factor for other diseases. The probability for a concomitant diagnosis of depression was discovered to be raised by a factor of 1.9 in persons with dementia, the probability of coronary heart disease by a factor of 3.1 and the probability of fractures by a factor of 3.1 [12]. Persons with Alzheimer disease were found to have an increased risk of acute illnesses (pneumonia), falls and hospitalisations in the United States [13] and in Germany [14]. A current study of primary care practices in Germany has yielded increased odds ratios for stroke (2.04) and depressive symptoms (1.36) in cognitively impaired persons [15]. Therefore, to deliver adequate treatment and care of persons with dementia, appropriate treatment of blood pressure, diabetes and a large array of other chronic diseases is of utmost importance. This already implies that treatment and care for people with dementia usually require multiprofessional approaches.

There are already many professions involved in the provision of adequate, guideline-oriented, high-quality care to persons with dementia. GPs typically know their patients for a long time and have developed a good relationship with them, which facilitates treatment. Specialists such as psychiatrists, neurologists and neuropsychologists deliver high-quality diagnoses as well as specific advice regarding, for example, pharmacological treatment. In the course of the illness, qualified nursing care becomes more important. In Germany, there is considerable heterogeneity in ambulatory services at inpatient and outpatient facilities that take care of persons with dementia. Different therapeutic approaches aim to maintain or even increase everyday functioning and mobility. Self-help groups for caregivers and caregiver counselling are another important element to support those who care for the patient at home. Even though this enumeration is not complete, it clearly indicates the complexity of the healthcare system for persons with dementia and their caregivers in Germany. Few of them, however, have ever been studied with regard to effectiveness.

Accessibility and utilisation of most of these options, however, is very much dependent on individual, systemic and regional variables. There is considerable need for better integration of care for patients with dementia. The psychiatric health care system in Germany includes all aspects of prevention, treatment, rehabilitation, care and research as well as education, but these areas have evolved rather separately, so that presently systematic coordination is lacking. This makes appropriate care for people with dementia even more difficult. [16] An adequate coordinated management of dementia care is urgently needed.

Relatives very often carry the highest burden of care for individuals with dementia who live at home. The existence of a caregiver at home is a prerequisite for good home-based care, at least when the patient is in the later stages of dementia. This role is usually taken by spouses or children [17,18]. A systematic review of health consequences for the caregiver showed that providing care to relatives with dementia is correlated with negative health outcomes for the caregiver, which often lead to an earlier institutionalisation of the person with dementia [19]. This relation is dependent on the age and sex of the caregiver, the relation to the person with dementia and other individual variables regarding the caregiver, including culture, coping strategies and other personal characteristics [19]. A recent Cochrane review indicated that there is scientific evidence for the efficacy of programs to reduce caregiver burden [20]. However, this conclusion was based on very few high-quality intervention studies. Therefore, population-based studies need to specifically consider the caregivers as much as the dementia patients themselves. More comprehensive, integrative, multiprofessional approaches may have the potential to improve the health of the caregiver as well as the person with dementia and to generate economic benefits within the healthcare system [21,22].

### Collaborative care in dementia

Trials in which researchers have studied collaborative care for dementia patients in the primary care setting have demonstrated its effectiveness [23-25]. However, these studies have been conducted outside Germany in different healthcare systems, so it is unclear whether these results extend to the specific context of the German healthcare system.

A GP-based, cluster-randomised trial for the improvement of healthcare provided for people with dementia and their caregivers in primary care Germany [26,27] was effective in improving referral rates to specialists. Part of the intervention was the recommendation of support groups and family counselling. The recommendation has increased the utilisation of support groups and counselling five- and fourfold, respectively. Utilisation of other support services remained low (less than 10%), with the exception of home nursing and institutional short-term nursing. However, we expect that actively managing care will have a greater effect on the healthcare of persons with dementia and their caregivers.

### Objective

The objective of this population-based intervention trial in the primary care setting is to test the efficacy and efficiency of implementing a subsidiary support system on a population level for persons with dementia who live at home. This subsidiary support system is initiated and coordinated by a dementia care manager (DCM): a nurse with dementia-specific qualifications. The main goals of this support system are (1) to improve quality of life, (2) to reduce caregiver burden, (3) to reduce behavioural and psychological symptoms of dementia and (4) to optimise pharmacotherapy with an antidementia drug and prevent the prescription of potentially inappropriate medication (PIM).

## Methods and study design

### The DelpHi cohort

**Figure 1:**
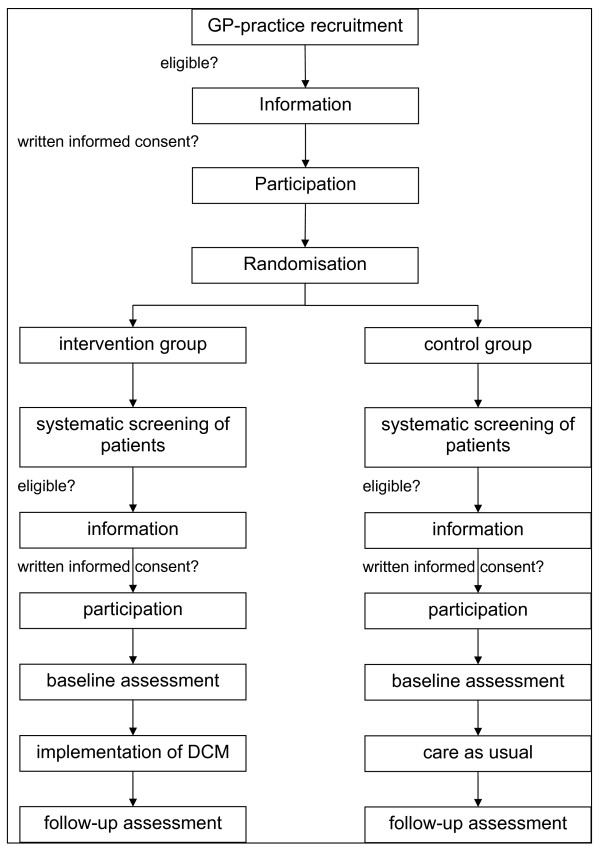
Flowchart of the DelpHi Trial.

The DelpHi Study has been designed to assemble a GP-based epidemiological cohort of people older than 70 years of age who live at home (the DelpHi cohort), from among whom we will identify those eligible to participate in a trial of dementia care management (the DelpHi Trial). (see the flowchart of the Delphi Study in Figure 1.) Participants in the DelpHi Trial will be followed prospectively and will be asked to participate in other trials in the future. Participants will be recruited from GP-based practices only; no other facilities will be used to identify eligible persons. All persons identified by a participating GP to be eligible for the study will be screened for cognitive impairment. People who meet the inclusion criteria will be informed in detail about the DelpHi Trial by the GP and will be asked for their participation. All persons who provide their written informed consent to participate will then be included in the DelpHi Trial.

This protocol describes the methodology for identifying and recruiting participants for both the cohort study and the trial parts of the DelpHi Study and the design and intervention of the DelpHi Trial.

**Figure 2:**
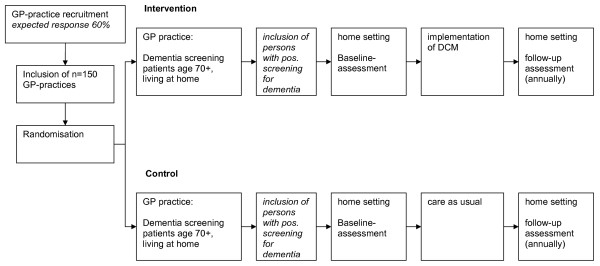
Design of the DelpHi Trial.

The trial has been designed as a GP-based, randomised controlled intervention trial to last for 48 months, comprising a 24-month baseline assessment, an overlapping 24-month intervention period and an annual follow-up assessment. The study design is illustrated in Figure 2.

### Identifying participants

GP practices participating in the DelpHi Study will systematically screen patients ages 70 years and older in their routine care. In cases where the screening instrument indicates dementia, the persons are eligible for the DelpHi Trial and will be informed in detail about the trial and invited to participate in it. Participants will be asked to name a caregiver who will be asked to participate in the trial as well. As cognitive decline is a core feature of dementia, the GP must evaluate the ability of the patient to provide his or her informed consent. If the screened person is not able to give informed consent, the caregiver will be asked to do so. This procedure has been discussed in detail and has been approved by the Ethical Committee of the Chamber of Physicians of Mecklenburg-Western Pomerania (registry number BB 20/11).

### The DelpHi trial

Participants and their caregivers will be cluster-randomised into one of two groups: (1) a DCM group (DCM, intervention group) or (2) a care-as-usual group (control group). GP practices represent the clusters for randomisation. All participants will be contacted by study staff to arrange a baseline assessment at the persons’ homes.

Participants randomised to the intervention arm will be contacted by a DCM to arrange the first home visit during the initial part of the study. Over a period of 12 months, the DCM will be in contact at least monthly with the participant; depending on the individual participant’s needs, more frequent contacts might be needed within the first 6 months of the trial.

Participants randomised into the control group will receive no specific intervention. These participants will be visited at home for a comprehensive baseline assessment but will receive care as usual (care-as-usual group).

Because persons with dementia are supposed to benefit from this trial, special emphasis will be put on defining and classifying participants on the basis of their dementia syndrome. Because a screening test at the GP practice is not sufficient and will yield false-positive results, a comprehensive assessment of all participants in the control and intervention groups will be conducted.

### Trial intervention

#### Intervention group: role of dementia care manager

Participants randomised to the intervention group will receive improved integrative and collaborative care involving different professions where needed. Their care will be coordinated by a DCM who is specifically trained in dementia care management. Their GP will receive comprehensive reports and be closely involved in the participant’s care. A more detailed description of the concept of a DCM, including qualifications and implementation, has been given elsewhere [28].

The DCM, in close cooperation with the GP, will establish and maintain a comprehensive subsidiary support system for persons with dementia and their caregivers. The overall goal of the intervention is to optimise access to and provision of healthcare for people with dementia and their caregivers. The DelpHi intervention is complex, multidimensional and multimodal and will be individually tailored to each participant and his or her ambient and social contexts and resources. The starting point is a detailed and systematic notebook-style, computer-assisted assessment conducted by the DCM of the needs of the person with dementia and the caregiver. Predefined algorithms suggest specific actions and treatment.

The intervention can be conceptualised as standing on three pillars: (1) treatment and care management, (2) medication management and (3) caregiver support. In improving the person’s situation, the DCM will systematically assess the resources and needs in eight action fields: medical diagnostics and treatment, nursing care and treatment, nonmedical therapies, social inclusion and/or support, legal counselling, technical assistance and telemedicine, pharmacological treatment and care, and caregiver support and education.

The intervention will be delivered according to a detailed protocol. The DCM will meet the person with dementia and the person’s caregiver for the baseline assessment and upon the first interventional visit, usually at the participant’s home. Further mandatory personal contacts will then be scheduled monthly for the first 6 months of the intervention and by telephone for the last 6 months of the intervention period. In addition to these mandatory contacts, optional contacts will be possible during the first 6 months. Optional contacts can be made in person or by telephone, depending on the person’s individual needs and preferences.

The personal resource and needs assessment will be analysed by the DCM, and a summary will be forwarded to the person’s GP. Treatment paths and specific actions will be discussed and implemented in close cooperation with the GP.

### Control group

Participants cluster-randomised to the control group will receive care as usual in a primary care setting.

### Inclusion and exclusion criteria

Eligible participants will be identified from among the patients of the participating GP practices. The inclusion criteria are that the person must be at least 70 years of age, living at home, have screened positive for dementia (score 8 or lower) on the DemTect Scale [29,30] and meet none of the exclusion criteria. The exclusion criteria are insufficient German-language competence and other medical conditions that do not allow testing (for example, hearing impairment, visual impairment).

### Sample size calculation

The estimated enrolment for the study is 1,000 participants and their respective caregivers. This sample size is necessary in the context of a complex intervention which could not be proven efficacious in smaller cohorts. To detect small effects (Cohen’s *d* = 0.2) in comparisons between intervention and control conditions at a significance level of α = 0.05 and a statistical power of 80%, a sample size of *n* = 310 persons per group would be needed. With the longitudinal design we calculate with a “loss due to follow-up” of 35%, we end up with *n* = 477 persons per group with complete data sets. According to the classification of Cohen, this would yield enough statistical power to detect small effects [31].

The required sample size can decrease during the course of the study if effects on the primary outcomes should be detected that are, on average, larger than anticipated in this calculation or if the loss during follow-up is smaller. If the average effects of this complex intervention on any relevant variable should be smaller, however, the sample size required to statistically significantly demonstrate them would be larger.

For example, an efficacy study for the implementation of a collaborative care model for older adults with Alzheimer disease in primary care [25], the primary outcome, behavioural symptoms measured with the Neuropsychiatric Inventory (NPI) [32], yielded small to medium effect sizes between Cohen’s *d* = 0.4 and Cohen’s *d* = 0.68 (our own calculations based on [32]). For several reasons, however, it is questionable whether these results are generalisable to Germany. First, the quality of health care in Germany is different from that in the United States. The cited study was conducted with an underserved population, whereas the DelpHi Trial will be conducted with people who benefit from a high-quality healthcare system. This difference will decrease the anticipated effect size. Second, the study itself will increase healthcare above the level of usual care in the control group because of the comprehensive assessment at home and in the GP practice. We can control for these factors; however, they will decrease effect sizes, and therefore we calculated our sample size for the conservative assumption of achieving a small effect.

The sample size will decrease over time for various reasons. Loss to follow-up due to death, migration, institutionalisation and decline to consent to participate will occur. Migration is unlikely to have any larger effect, because the persons older than 70 years of age tend to be geographically stable. When a participant is lost to follow-up, efforts will be made to locate and recontact the participant. The data will be included in the main analysis. Decline of initial consent will be documented whenever possible, together with the reasons provided. Mortality and institutionalisation will be captured by regular visits to the GP and in the course of the follow-up visits at home.

### Recruitment

Recruitment will take place in the GP practice on the basis of the inclusion and exclusion criteria listed above. Participating GPs will systematically screen their patients at regular GP visits. Inclusion criteria for GP practices are that they provide primary care in a residential practice in Mecklenburg-Western Pomerania. A list of eligible practices is provided by the chamber of physicians of Mecklenburg-Western Pomerania. Eligible GP practices are contacted by sending an information letter about the study and a personal visit by study staff to inform GPs face-to-face about the study and all associated procedures. Upon providing their written consent to participate in the study, the GP practices are randomly assigned to either the intervention or control group. Potential participants with dementia who meet the inclusion criteria will be identified by the GP.

If the screening test is positive, the GP will provide written and oral information about the study to the person with suspected dementia. Because the caregiver is an equally important target of the study, too, the person with dementia will be asked to provide contact details of his or her caregiver, who then is also informed about the study, usually by the GP. Providing a caregiver is not an inclusion criterion for the study and can be denied by the person with dementia. Nevertheless, it is our goal to recruit caregivers for a large proportion of the persons with suspected dementia. Because cognitive decline is a symptom of dementia, the GP has to assess whether the person with dementia is capable of giving valid written informed consent. In cases in which the person is incapable of providing consent, written informed consent is obtained from the caregiver. The GP collects the written informed consent forms of the person with dementia and the caregiver. The forms are regularly retrieved from the GP practice by study staff. Anonymised information regarding the result of the screening test and the patient’s age and sex is collected from participants who decline to participate in the trial to allow for some basic comparisons between them and the participants in the study. The GP receives an incentive of 10€ per screening and 100€ per written informed consent.

As part of the baseline assessment, the study team will assess the syndrome of dementia by conducting a structured interview for the diagnosis of dementia according to the International Classification of Diseases, 10th Revision, or ICD-10 [33]. This will help identify participants who were screened false-positive. It also helps to define the participants recruited into the study according to the different syndromes of dementia. Participants without a syndrome of dementia (that is, false-positive) will be included in the follow-up assessment but excluded in calculating the study outcome.

### Randomisation

Participating GP practices that meet inclusion criteria and provide written informed consent are randomised into the trial. Simple randomisation with a 1:1 allocation ratio is carried out by fair coin-tossing right after the study team has received the written informed consent form from the GP. Simple randomisation is recommended by the CONSORT group because it “is elegantly sophisticated in that it is more unpredictable and surpasses the bias prevention levels of all other alternatives” (http://www.consort-statement.org/consort-statement/further-explanations/box2_randomisation_minimisation/). However, coin-tossing in itself is not recommended, but was chosen for several reasons. There is evidence that simple coin-tossing will lead to problematic sample sizes in small clinical trials (*N* < 100) but that it can be trusted to generate equal numbers in large trials (*N* = >200) [34]. It is also well-known that “[p]roper allocation concealment frequently frustrates clinical inclinations, which annoys those who do the trials.… Many involved with trials will be tempted to decipher assignments, which subverts randomisation” [35] (p. 614). In our study, GPs will be randomised after they have given their written informed consent. This will prevent the possibility that the initial recruitment of GP practices might be biased by knowledge of the allocation into the intervention and control groups. Stratified randomisation would be difficult because of the continuous process of enrolling GPs during the trial. Block randomisation would induce problems due to limited information on the covariates to be controlled for [34].

### Follow-up

Data will be collected annually throughout the study. Data will be collected by the GP practices (during the screening assessment) at baseline at the person’s home (notebook computer-assisted face-to-face interviews). After inclusion of a person and based on explicit informed consent, secondary data from the person’s medical record will be abstracted at the GP practice at baseline and will be repeated annually. The same assessment instruments will be used for the intervention and control groups.

#### Outcome measures

The primary outcomes in this complex intervention trial are quality of life and healthcare for patients with dementia and their caregivers. These are multidimensional outcomes with a focus on four dimensions: (1) quality of life, (2) caregiver burden, (3) behavioural and psychological symptoms of dementia and (4) pharmacotherapy with an antidementia drug and prevention or suspension of PIM. Quality of life will be measured using the Quality of Life in Alzheimer’s Disease instrument [36], which consists of 13 items and includes “assessments of the individual’s relationships with friends and family, concerns about finances, physical condition, mood, and an overall assessment of life quality” [37] (p. 55). To measure caregiver burden, the Berliner Inventar zur Angehörigenbelastung (BIZA-D) [38] will be used. BIZA-D is a standardised, theoretically grounded, psychometrically validated instrument used to assess burden and stress. It covers physical exhaustion, personal restrictions in life, missing social appreciation, personal development and negative appraisal. It also assesses tasks required in caring for the person with dementia, in motivating and guiding, in supporting in care, in emotional support and in oversight. The Neuropsychiatric Inventory (NPI), developed by the Alzheimer’s Disease Cooperative Study investigators [32], is used as a standardised instrument to assess behavioural and psychological symptoms. The assessment of pharmacotherapy with antidementia drugs focuses on the following substances which are approved by the drug authorities and recommended by the current guidelines [39,40]: donepezil, galantamine, rivastigmine and memantine. Additionally, we evaluate the reduction of PIM according to the PRISCUS criteria [41] as well as the reduction of anticholinergic drugs [42].

The following are the secondary outcomes:

1. The Structured Interview for the Diagnosis of Dementia, or SIDAM [33], will serve to identify screening false-positives as well as to differentiate different syndromes of dementia.

2. Activities of daily living will be assessed using the Bayer Activities of Daily Living Scale, or B-ADL [43,44].

3. Social support will be assessed using the *Social Support Questionnaire (*F-SozU) [45].

4. Health status will be assessed using several instruments that measure health-related variables of the person with dementia, including the GP records, the SF-12 Health Survey [46], the standardised assessment for elderly patients in a primary care setting (STEP) [47], the Brief Symptom Inventory (BSI) [48] and the Gesundheitsfragebogen für Patienten (PHQ-D) [49,50].

5. Utilisation of health care resources will be assessed according to GP and specialist visits, outpatient treatments, inpatient treatments, hospitalisations, nursing home admissions, therapeutic appliances and provision of informal care [51].

6. Medications will be assessed by the DCM, who will conduct an information technology-supported standardised home medication review [52] at the patient’s home with subsequent medication management by the patient’s local pharmacy regarding the frequency of drug-related problems, intake of PIMs, clinically relevant drug-drug interactions, adherence, adherence to supportive activities (that is, medication plan, drug dispenser, support by care service, reduction of the number of drugs taken and home medication review) [52].

#### Data analysis

##### Statistical analysis

Logistic regression analysis will be conducted to compare the intervention and control groups regarding their respective average scores on the primary outcome measure. Secondary analyses will be conducted using linear or logistic regressions, depending on the outcome under analysis.

A randomisation check will be conducted by comparing the intervention and control groups on various variables at baseline. In case of any differences, these variables will be adjusted for in the analysis of the outcomes.

Descriptive analyses will be conducted to evaluate the characteristics of the participants at baseline and follow-up. Response rates will be calculated for each group at each time point of analysis and compared between groups. In univariate and bivariate analyses, the number of missing data will be identified. In multivariable analysis, imputations will be based on the group averages. Appropriate sensitivity analyses will be conducted.

#### Withdrawal

Participation in the study is voluntary, and withdrawal can occur at any time. Upon withdrawal, data will be anonymised. Anonymised data will be retained unless a person specifically requests that his or her data be physically deleted.

#### Economic analysis

In the economic analysis, we will investigate the efficiency of the intervention in comparison to controls who received usual care. Efficiency will be quantified as ratios of predefined clinical outcomes to costs. Therefore, both all relevant outcomes (primary and secondary) and all direct costs (dementia care management and utilisation costs) will be captured and documented throughout the trial. Furthermore, incremental cost-effectiveness ratios will be calculated from different viewpoints (society, payer and patient), and relevant sensitivity analyses (multiway, scenario-based and probabilistic) will be conducted. During the course of the trial, detailed cost-monitoring of the direct care management intervention will be carried out to identify key factors determining cost-effectiveness that can be used to inform future dementia care.

#### Ethical issues

Ethical approval for this trial has been obtained from the Ethical Committee of the Chamber of Physicians of Mecklenburg-Western Pomerania (registry number BB 20/11).

An ethical issue specific to this study is the capability of persons with dementia to give informed consent. In accordance with the Ethical Committee’s recommendation, we assigned to the screening GP the decision regarding a person’s capability of providing consent. Information about dementia and assessing the person’s capability to consent are routine tasks for GPs in primary care. In cases of incapability, the caregiver will be asked to provide written informed consent for the participation of the person with dementia.

During the course of the study, it is likely that the person with dementia will lose the capability to consent because of cognitive decline. The study staff will regularly check with the GP whether written informed consent needs to be obtained by the caregiver to keep the person with dementia in the study. This will be done and documented prior to any follow-up assessment.

#### Trial management

The principal investigator (WH) will be in charge of the overall management of the trial. The scientific coordinator (JRT) will be responsible for the coordination of the trial. A nursing scientist (AD) will be responsible for the qualification and quality assurance of the DCM. Study staff will carry out the day-to-day activities involved in running the trial. Delivery of the intervention will be carried out by specially trained and qualified nurses. A psychiatrist (GB) will be responsible for treatment and care management (intervention pillar 1). A pharmacist (TF) will be responsible for medication management (intervention pillar 2). A psychologist (JRT) will be responsible for caregiver support (intervention pillar 3). Members of the study team will convene weekly to ensure compliance with the study protocol and to ensure a high quality of intervention throughout the study.

### Data quality

The data collection process will be based on computer-assisted personal interviews. All data are entered by the DCM into electronic case-reporting forms. The DCM will use a Java-based rich-client platform which is run on a mobile touch-screen tablet PC. MySQL database software will be used for data storage. The tablet personal computer connects to the study server through designated cradles using virtual private network technology. The client server automatically stores all data. Special encryption procedures ensure state-of-the-art data safety. The DCM can print predefined standardised reports for communication with the GP and the participating pharmacist by using portable printers with Bluetooth access.

The DCMs will receive intensive training in data collection. The baseline training takes approximately 40 hours. Every 3 months the DCMs will undergo a refresher module (4 hours) to ensure data quality. The DCMs are prompted upon entering implausible data.

All processes concerning data assessment, management and preparation for analysis will be defined according to comprehensive standard operation procedures (SOPs). SOPs are updated every 6 months. Currently, SOPs address the following topics:

1. The conduct of dementia screening in the GP practice

2. The first contact with the patient

3. The baseline assessment in the patient’s household, including application of the software

4. Personal hygiene issues

5. The standardised intervention

6. Medication management by the local pharmacist

7. Data management standards

A scientific advisory board composed of members independent from the trial and the funding organisations was elected. Members include dementia experts in various fields of research (clinical psychiatry, nursing science, family medicine, social politics, intervention research, epidemiology and so on) and meet once yearly.

#### Expected results

We expect to find statistically significant differences between the intervention and control groups across all primary outcome measures. Specifically, persons with dementia and their caregivers in the intervention group will have a higher quality of life than those in the control group. There will be less caregiver burden and less behavioural and psychological symptoms of dementia in persons of the intervention group. The medical treatment with antidementia drugs will be more frequent and treatment with PIMs will be less frequent than in the control group. These effects will be dependent on various variables, including age, marital status, morbidity, severity of dementia, cognitive status, activities of daily living at baseline and others.

### Adverse events

We do not expect adverse events to occur during our trial. Treatment and care will be based on current guidelines and supervised by and coordinated with the treating GPs in routine care. There will be no pharmacological interventions by the study staff. The study staff are specifically qualified for the tasks associated with the conduct of the intervention.

As in other clinical trials, some adverse events are possible. One aspect concerns psychological symptoms which may occur as a result of our questions. The DCM will receive intense training by the psychiatrist to handle adverse events. An emergency plan is one part of the baseline SOPs. During home visits, a telephone contact with the GP as well as with the trial psychiatrist is possible. In weekly case conferences, experiences in the household will be discussed and processes will be adjusted where appropriate.

## Discussion

### Interpretation

We expect the results to be interpreted as supportive evidence for a positive impact of the tasks of a DCM in the primary care setting. The results will provide evidence for specific needs in ambulatory care of persons with dementia and will show effective ways to meet those needs. Thus the study will yield information regarding how to better manage current problems in routine dementia care. Qualification requirements for DCM will be evaluated. The results of the study will help to modify existing guidelines and treatments and develop new ones.

### Generalisability

Because of the real-life settings in which the trial will take place, the results will be readily generalisable to the primary care setting in Germany. The study area at this stage is confined to only one federal state. However, the legal base, the structures, and the intrinsic organisation of the primary care system in Germany are identical across all German states. With Mecklenburg-Western Pomerania being a rural area in an industrialised nation struck by demographic change and facing dementia care as one of the upcoming challenges of the near future, the results will also elucidate implications for regions facing similar challenges across the world.

## Trial status

Enrolment into the study started on 1 January 2012.

## Abbreviations

DCM: Dementia care manager; DelpHi: Life- and person-centred help in Mecklenburg-Western Pomerania: Germany; GP: General practitioner; SOP: Standard operation procedure.

## Competing interests

The authors declare they have no competing interests, with the exception of HJG and HF. HJG has received funding from the German Research Foundation and the Federal Ministry of Education and Research Germany. He has received speaker’s honoraria from Bristol-Myers Squibb, Eli Lilly, Novartis, Eisai, Boehringer Ingelheim and Servier, as well as travel funds from Janssen-Cilag, Eli Lilly, Novartis, AstraZeneca, Lundbeck and SALUS-Institute for Trend-Research and Therapy Evaluation in Mental Health. HJF has received financial support from the German Research Foundation; the Social Ministry of the Federal State of Mecklenburg-West Pomerania; the Family Ministry of the Federal Republic of Germany, as well as speaker’s honoraria from AstraZeneca, Eli Lilly and Novartis and travel funds from Janssen-Cilag.

## Authors’ contributions

All authors contributed to the design and development of the study protocol in their area of special expertise. JRT drafted the manuscript, is the coordinator of the study and has substantially contributed to the overall design of the study. TF is responsible for the pharmaceutical aspects of the study design. AD focuses on the quality of dementia care management and quality of qualification in dementia care management. GB developed the psychiatric part of the interventions. AA developed the medical part of the interventions. SL developed the design of the health economics analyses. ST influenced the medical interventions as well as the psychiatric assessments in the study. SF supervised the health economics analyses and wrote the corresponding parts of the manuscript. HJF and HJG developed the psychiatric assessments and interventions. WH is the principal investigator of the study, has contributed substantially to the concept of the study and wrote the manuscript. All authors read and approved the final manuscript.
